# A Guide to Developing a Neurosurgery Quality Dashboard From Scratch: A Technical Report

**DOI:** 10.7759/cureus.113035

**Published:** 2026-07-20

**Authors:** Arshad Ali, Chithra Joseph

**Affiliations:** 1 Department of Clinical Academic Sciences, Qatar University, Doha, QAT; 2 Department of Neurological Sciences, Weill Cornell Medicine, Doha, QAT; 3 Department of Neurosurgery, Hamad General Hospital, Doha, QAT; 4 Department of Health Professional Education, School of Graduate Studies, University of Maryland, Baltimore, USA; 5 Department of Nursing, Hamad Medical Corporation, Doha, QAT

**Keywords:** care bundles, neurosurgery, patient safety, performance metrics, quality dashboard, quality improvement, resource-limited setting

## Abstract

Measurement is the foundation of quality improvement, yet many neurosurgical departments, particularly those early in their quality journey or with limited informatics resources, lack a structured way to track their performance. Most published neurosurgery dashboards originate from well-resourced academic centers, rely on electronic medical records or business intelligence platforms, and are described only after they have matured. We report the design and implementation, from first principles, of a manually maintained neurosurgery quality dashboard at the Neuroscience Institute, Hamad Medical Corporation, Doha, Qatar, during 2021, at the height of the COVID-19 pandemic. The dashboard was organized into eight quality bundles (Volume, Timely Discharge, Throughput, Service, Clinical Care, Patient Satisfaction, Infection Control, and Patient Safety), each defined in advance by its component indicators, numerator and denominator, data source, and sampling frame. A multidisciplinary team was formed; each bundle was assigned a named frontline owner and a backup; data were collected on standardized spreadsheets, compiled monthly into charts, and reviewed by the team to guide action. We present one year of data as a worked illustration. The dashboard surfaced several actionable signals, including a low rate of discharge before noon, most often attributed to delayed physician orders; episodic transfer-out delays; suboptimal glycemic control; higher physician-domain satisfaction scores in the second half of the year than in the first; consistently high hand-hygiene compliance with no device-related infections; and a low absolute burden of safety events. These observations are descriptive and uncontrolled, were not statistically tested, and are presented to illustrate the dashboard rather than to establish cause or effect. The experience shows that a functional, action-oriented neurosurgery dashboard can be built without advanced informatics when its metrics are clearly defined, its responsibilities are assigned, and its review is disciplined. We offer the eight-bundle structure as a template for departments beginning a quality program, including in resource-limited settings.

## Introduction

"If you can measure it, you can manage it." In modern healthcare, quality must be measured before it can be understood, managed, or improved. The idea is not new. It traces to Ernest Codman's early twentieth-century insistence on the "end result," the disciplined follow-up of every patient to learn whether treatment truly helped [1], and it acquired fresh urgency with the Institute of Medicine's To Err Is Human, which reframed preventable harm as a property of systems and called for care that is safe, effective, patient-centered, timely, efficient, and equitable [2]. Neurosurgery, where the margin between benefit and catastrophe is narrow, has a particular stake in this agenda [3-5].

From this imperative grew the quality dashboard, in which a curated set of indicators is displayed together so that performance can be seen, interpreted, and acted upon. The University of California, Los Angeles, was among the first neurosurgical departments to formalize the approach, organizing its dashboard around quality and safety, patient satisfaction, and efficiency and use [3,6]. Others standardized outcomes nationally through the N2QOD/NeuroPoint registry [7], built condition-specific dashboards [8], embedded indicator frameworks in national pediatric programs [9], and addressed discrete problems such as patient satisfaction [10], clinical documentation and length of stay [11], and unplanned reoperation [12], within the wider discipline of quality and risk management [13-15] and the safety culture that durable improvement demands [16].

Much of this experience, however, shares a common vantage point. On a focused, non-systematic reading, the accounts we identified came largely from well-resourced academic centers, presupposed electronic medical records or business-intelligence platforms, and described mature systems rather than the work of building one. We did not perform a systematic review and offer this as a narrative observation, but the scarcity of descriptions of how a department builds a dashboard from scratch, manually, and with limited resources is the gap we address. The pressing question for such departments is not how a sophisticated dashboard performs, but how one begins: what to measure, who collects the data, and how disciplined review is sustained with only a spreadsheet and committed staff. This Technical Report answers that question. We describe a neurosurgery quality dashboard built from first principles, organized into eight bundles, and maintained manually by a multidisciplinary team through the COVID-19 pandemic. We offer its structure as a template that other departments, including those in resource-limited settings, may adopt or adapt.

## Technical report

Setting and baseline context

The project was undertaken at the Neuroscience Institute of Hamad Medical Corporation, a tertiary referral center in Doha, Qatar, whose three departments of Neurology, Neuroradiology, and Neurosurgery together deliver the full range of neuroscience care. The Neurosurgery department began this work without any structured means of measuring its own performance. Quality was discussed but not counted, and decisions rested more on impressions than on evidence. The dashboard described here grew out of a deliberate decision to close that gap, undertaken by a team that openly regarded itself as a beginner in the formal discipline of quality improvement. The work was carried out through 2021, at the peak of the COVID-19 pandemic, a period of acute staffing shortages and system-wide disruption that formed the demanding backdrop against which the dashboard had to function.

Building the quality team and governance

**Figure 1:**
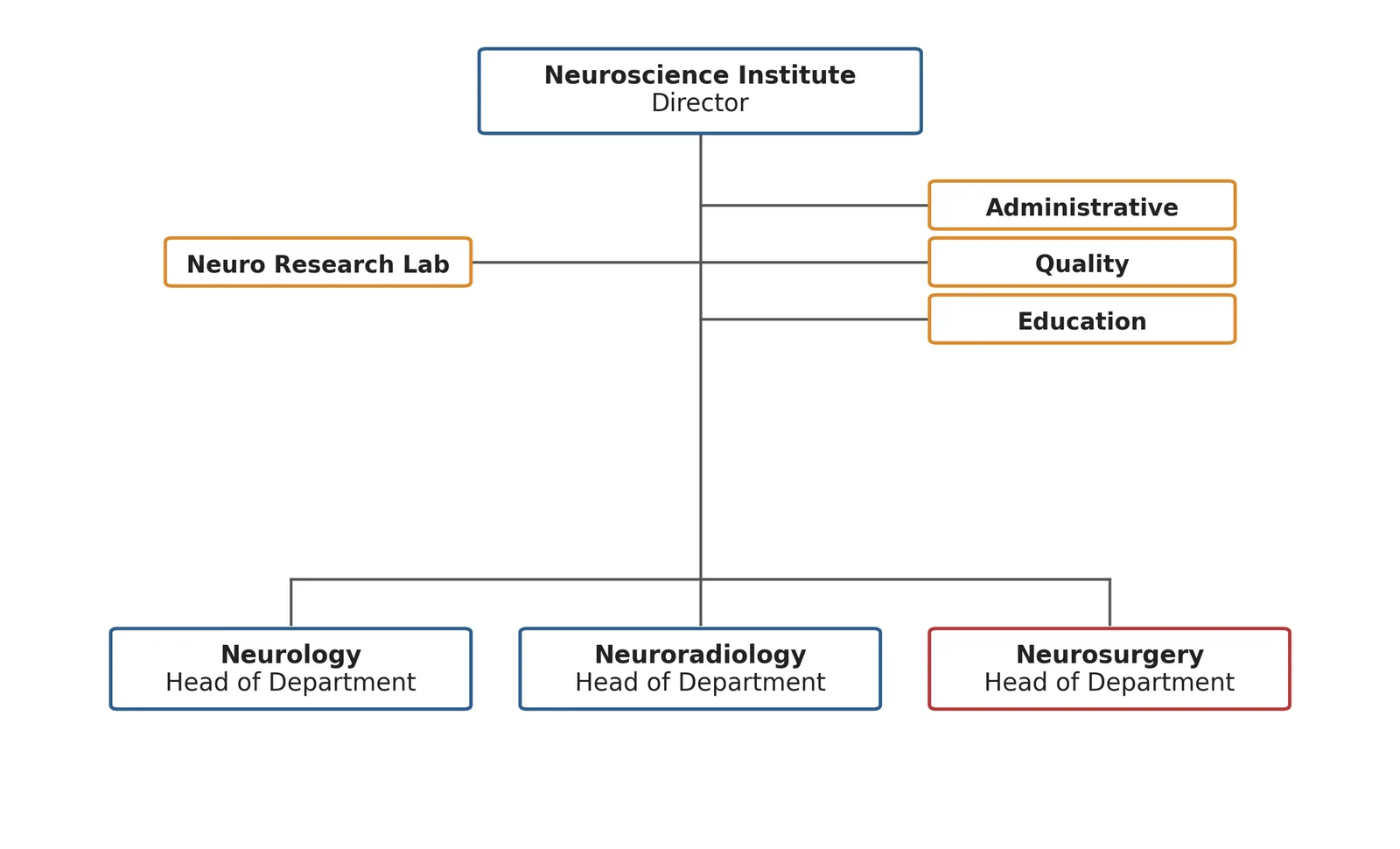
Organizational structure of the Neuroscience Institute and the Neurosurgery Quality Team Organogram of the Neuroscience Institute, Hamad Medical Corporation, showing the Director, the supporting Administrative, Quality, Education, and Neuro Research Lab units, and the three constituent departments (Neurology, Neuroradiology, and Neurosurgery). No artificial intelligence (AI) tool was used to generate the figure.

Measurement of this kind cannot be delegated to a single individual, and our first step was therefore to assemble a multidisciplinary quality team rather than to choose indicators. Its membership was chosen so that every function the dashboard would depend upon was represented: the head of neurosurgery and senior surgeons to provide clinical authority, the director of nursing and head nurse to connect the effort to the bedside, dedicated quality nurses to carry the daily work of data capture, infection-control practitioners, and a reviewer from corporate quality to align the project with institutional standards. This breadth secured leadership engagement, which previous experience identifies as a precondition for a dashboard's success [1]. The resulting structure is shown in Figure 1.

Selecting the metrics: an eight-bundle architecture

Having formed the team, we turned to the question of what to measure, and chose to group related indicators into eight quality bundles rather than to track isolated numbers. A bundle gathers a small set of individually measurable components under a single theme, making the work easier to assign, collect, and review, and allowing our local priorities to sit comfortably within the broad domains used by established programs [6,9]. The eight bundles were Volume, Timely Discharge, Throughput, Service, Clinical Care (glycemic and hypertension control), Patient Satisfaction, Infection Control, and Patient Safety. For each, we specified its component indicators, the numerator and denominator wherever a rate was involved, the data source, and the sampling frame, and we fixed these definitions in advance so that the meaning of each measure would remain stable from month to month and from one data collector to the next. The full set of definitions is given in Table 1.

**Table 1:** The eight quality bundles: indicators, definitions, data sources, and sampling frames Definitions of the eight quality bundles comprising the neurosurgery quality dashboard, including each bundle's component indicators, numerator/denominator where a rate applies, data source, and sampling frame. Thirty-day readmission was defined in advance as a Service-bundle component but was not operationalized during the 2021 pilot year (see Results); it is retained here as a planned indicator to be captured as the program matures. OHW: other healthcare workers; SBP: systolic blood pressure; DBP: diastolic blood pressure; WHO: World Health Organization; EVD: external ventricular drain; MRSA: methicillin-resistant *Staphylococcus aureus; *CR-BSI: catheter-related bloodstream infection; CAUTI: catheter-associated urinary tract infection; PICC: peripherally inserted central catheter

Bundle	Component indicators	Numerator/denominator	Data source	Sampling frame
1. Volume	Elective, emergency, transfer-in, transfer-out admissions; total census	Counts	Admission/registry log	Full count
2. Timely Discharge	Discharge order before 10 AM, discharge before noon, total discharges, and delay reasons	N discharged before noon/total discharges	Discharge log + reason coding	Full count
3. Throughput	Transfer-in/out delays (hours), discharge delays beyond noon (hours)	Total delay hours/patients (hours/day)	Bed-management/transfer log	Full count of delayed events
4. Service	Bed occupancy, 30-day readmission, total mortality, sepsis mortality	Occupied/available bed-days, readmissions/ discharges	Registry + readmission coding	Full count
5. Clinical Care	Glycemic control (mean of three readings/24 hours), hypertension control (SBP ≤ 140, DBP ≤ 90)	N at target/N sampled	Nursing vitals/glucose chart	2/day -> 10/week -> ~40/month -> 480/year
6. Patient Satisfaction	11-item survey: physician, nursing, service-care domains + recommendation	N satisfied/N surveyed (per item)	Patient/family survey (English + Arabic)	2/day -> 10/week -> ~40/month -> 480/year
7. Infection Control	Hand-hygiene compliance (physician/nurse/OHW); MRSA, CR-BSI, CAUTI, PICC, EVD infections	WHO hand-hygiene tool; device-infection counts	Infection-control surveillance	Full surveillance
8. Patient Safety	Medication errors, falls, pressure injuries (stage 1/2), phlebitis	Counts per month	Incident reporting/nursing audit	Full count

Defining data collection

The means of collection were kept deliberately simple, in keeping with the resources actually available. Each bundle was provided with a standardized spreadsheet that specified precisely which fields to record, so that any team member could capture data reproducibly. Bundles that rested on complete operational counts, such as volume, discharge timing, throughput, service metrics, infection surveillance, and safety events, were drawn in full from existing registry and operational logs. Bundles that required direct patient-level assessment, namely clinical care and patient satisfaction, were sampled at a pragmatic rate designed to balance representativeness against the workload of frontline staff: two patients per day, 10 per week, roughly 40 per month, and a target of about 480 over the year. Patient satisfaction was assessed using a structured survey covering physician care, nursing care, and service care, along with an overall recommendation item, and it was administered in both English and Arabic to remain accessible to the patients it was meant to serve. Not all bundles began data capture on the same date. As a program built from scratch by a team new to quality work, individual bundles were operationalized as their data-collection workflows could be established. Structured glycemic monitoring within the Clinical Care bundle, in particular, was operationalized only in the second half of 2021 for logistic and technical reasons, including setting up the sampling workflow, coordinating structured glucose charting with nursing, and engaging endocrine services; its first-half values were therefore not prospectively captured.

Assigning roles and workflow

Accountability was made explicit. Every bundle was assigned a named frontline owner responsible for its day-to-day data capture, and a named backup to ensure continuity when staff were unavailable. In contrast, compiling data into charts and the final assembly of the dashboard were the responsibility of the project lead and head nurse. In practice, the frontline nursing staff owned most of the operational bundles. This arrangement distributed the effort sustainably and ensured that no measure depended on the availability of any one person.

Benchmarks and targets

It is important to be candid about targets. Formal numeric thresholds were not defined when the dashboard was launched; in its first year, the instrument served chiefly to establish baselines and to reveal trends. For this report, we have applied benchmarks retrospectively in a hybrid manner. Where a recognized external standard exists, we adopted it as a reference: hand-hygiene compliance of at least 80%, a level in common institutional use rather than a World Health Organization mandate, with the WHO observation methodology and multimodal improvement framework used to define and audit compliance [16]; bed occupancy of approximately 85%, blood-pressure control thresholds of 140 and 90 mmHg, a pre-meal glucose target of 6 mmol/L or below, satisfaction of at least 90% for each survey item, and an aspiration of zero harm for hospital-acquired infection and safety events. Where no such standard was available, as for throughput delays, discharge before noon, and volume, we used the first half of the year as an internal baseline against which the second half could be judged. The limitations of this retrospective approach, and our intention to set prospective targets as the program matures, are considered in the Discussion.

Compilation, display, and the review cycle

**Figure 2:**
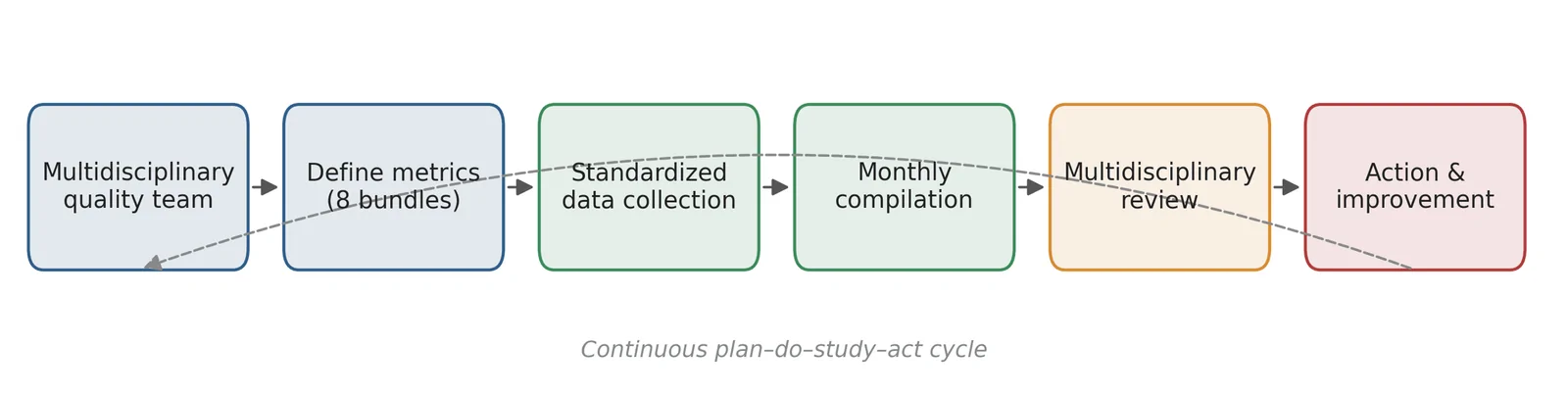
Workflow for developing and operating the neurosurgery quality dashboard End-to-end process showing formation of a multidisciplinary quality team, definition of the eight quality bundles and their metrics, standardized monthly data collection, compilation into charts, multidisciplinary review, and resulting improvement actions, operating as a continuous plan-do-study-act cycle. No artificial intelligence (AI) tool was used to generate the figure.

The dashboard was not an end in itself but the visible part of a recurring cycle. Each month, the owners' raw data were compiled into charts and consolidated into a single display, which the multidisciplinary team then reviewed, interpreting trends, identifying measures that were falling short, and agreeing on the actions that should follow. This monthly rhythm, which mirrors the cadence adopted by established neurosurgical dashboards [6], closed the plan-do-study-act loop [17] and allowed the dashboard to evolve through successive versions as its metrics and presentation were refined. The complete workflow is summarized in Figure 2.

Results

The Dashboard in Action

The data that follows were collected across the 2021 calendar year and are presented to show the kinds of signals the dashboard brought to the surface. The analysis is descriptive; its purpose is to demonstrate the instrument rather than to test a hypothesis.

Volume

**Figure 3:**
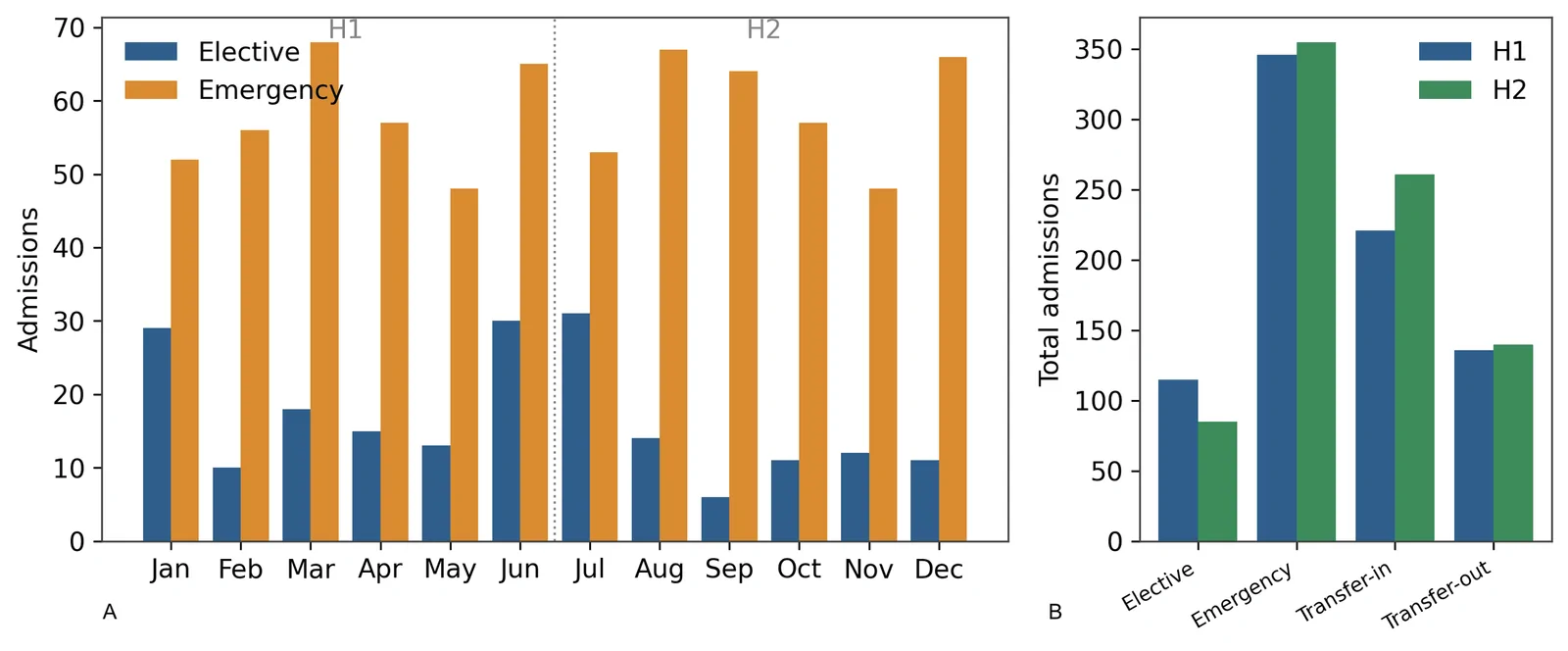
Volume bundle - neurosurgical admissions Monthly elective and emergency admissions across 2021 (A), and first-half (H1, January-June) versus second-half (H2, July-December) totals for elective, emergency, transfer-in, and transfer-out admissions (B). Emergency admissions predominated throughout the year, while elective admissions declined in the second half. No artificial intelligence (AI) tool was used to generate the figure.

Throughout the year, the surgical workload was dominated by emergency admissions. Elective admissions fell from 115 in the first half of the year to 85 in the second, while emergency admissions remained high, essentially unchanged at 346 and then 355. Transfers into and out of the unit rose modestly, from 221 to 261 and from 136 to 140, respectively. The contraction in elective work is consistent with the disruption that the COVID-19 pandemic imposed on scheduled care (Figure 3A-3B).

Timely Discharge

**Figure 4:**
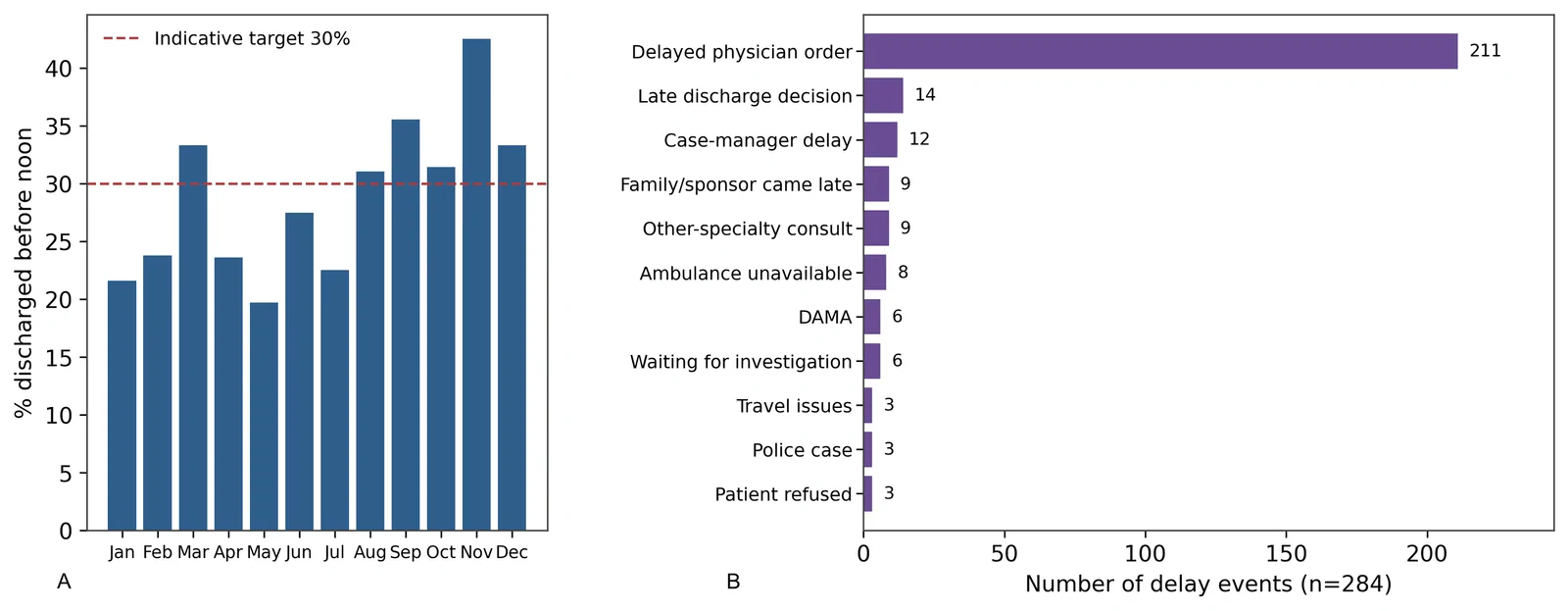
Timely-discharge bundle - discharge before noon and reasons for delay Monthly percentage of patients discharged before noon, with a 30% reference line applied retrospectively for orientation only (A), and a Pareto analysis of 284 discharge-delay events (B). Delayed physician discharge orders accounted for 211 events (approximately 74% of all delays). No artificial intelligence (AI) tool was used to generate the figure.

The proportion of patients discharged before noon remained low throughout the year, ranging from roughly 23 to 43% by month, highlighting timely discharge as a clear opportunity for improvement. A Pareto analysis of 284 delay events showed that a single cause, the late writing of physician discharge orders, accounted for 211 events, or about three-quarters of the total. In contrast, every other cause contributed only modestly. This concentration pointed directly to two interventions: accepting discharges based solely on physician orders and opening a discharge lounge (Figure 4A-4B).

Throughput

**Figure 5:**
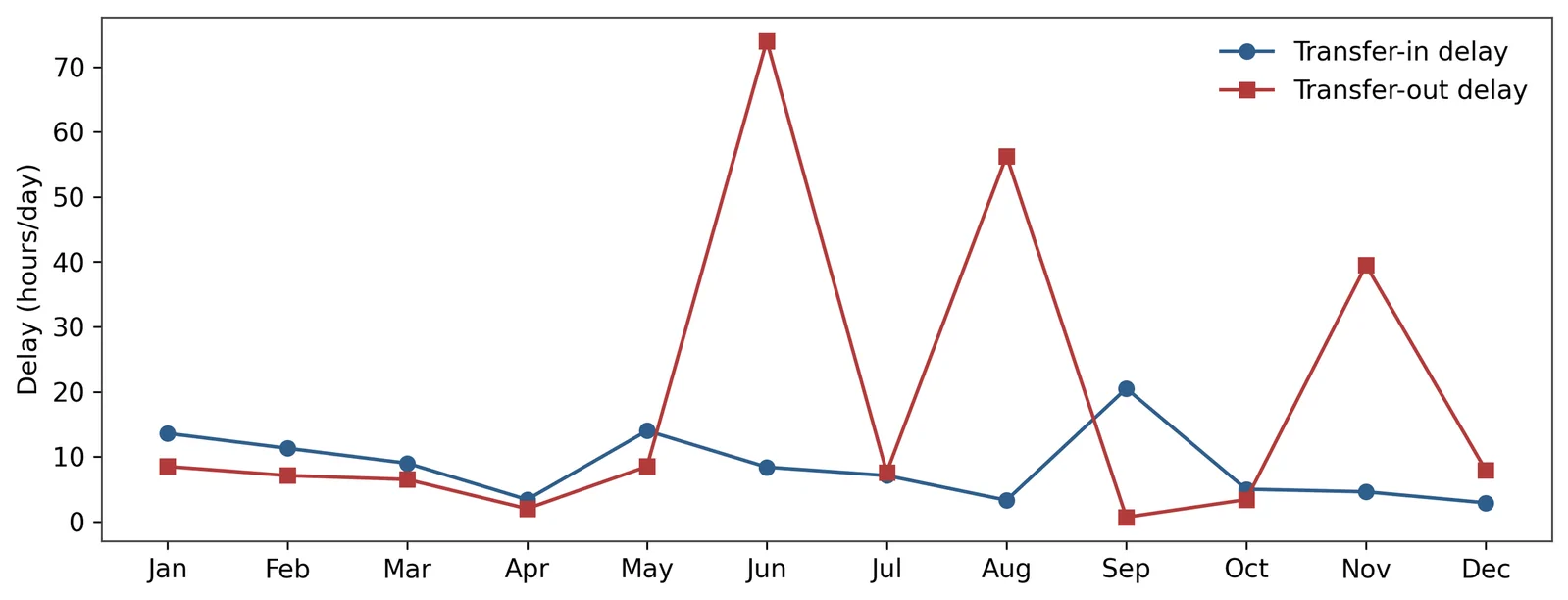
Throughput bundle - transfer delays Monthly transfer-in and transfer-out delays are expressed as delay-hours per day. Transfer-out delays showed pronounced episodic spikes in June, August, and November, consistent with intermittent downstream bed-availability constraints. No artificial intelligence (AI) tool was used to generate the figure.

Transfer delays, expressed as delay-hours per day, were generally modest for incoming transfers but showed pronounced, intermittent spikes for outgoing transfers, reaching 74 hours per day in June, 56 hours per day in August, and 40 hours per day in November. The pattern suggested episodic constraints in downstream bed availability rather than a persistent bottleneck (Figure 5).

Service

**Figure 6:**
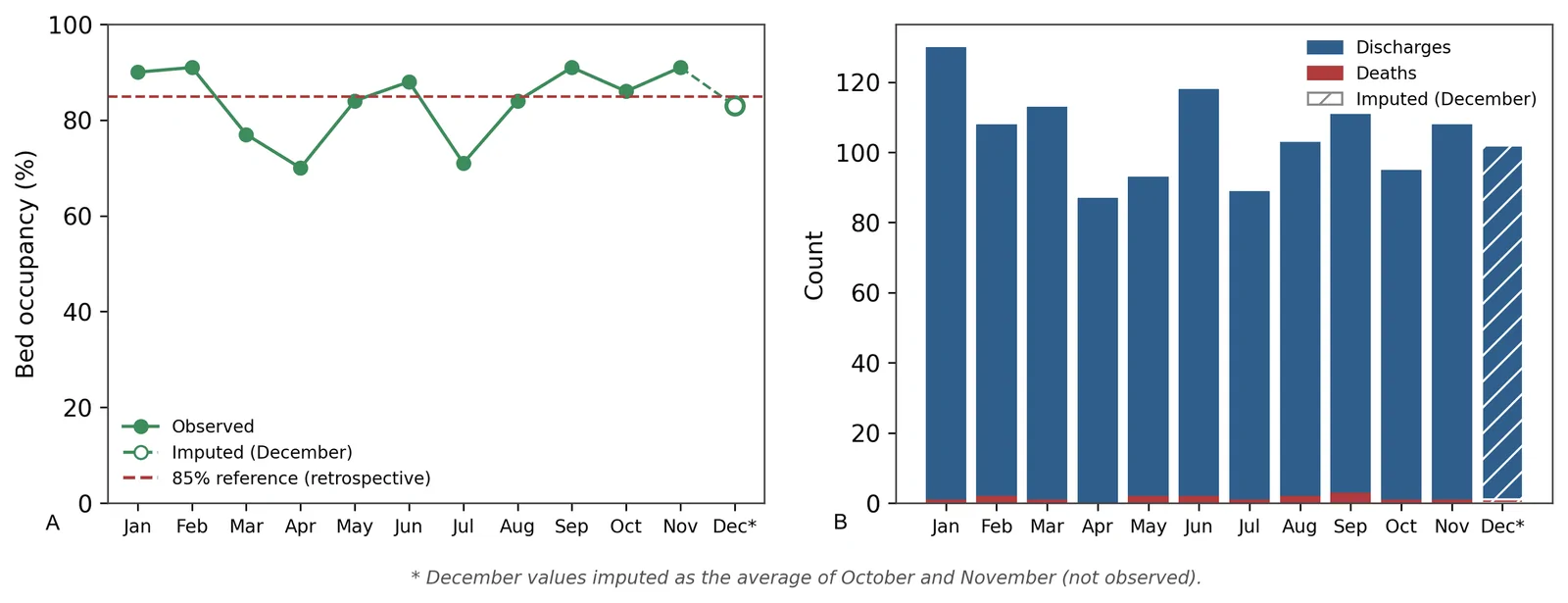
Service bundle - bed occupancy and mortality Monthly bed occupancy (A) and monthly discharges versus deaths (B). The December values (shown as an open marker on a dashed segment in (A), as hatched bars in (B), and marked with an asterisk) were imputed as the average of October and November and are not observed data. The 85% line in (A) is a reference applied retrospectively for orientation only. Sepsis-related mortality was zero throughout the year. No artificial intelligence (AI) tool was used to generate the figure.

Bed occupancy ranged from 70 to 91% over the year. Monthly discharges were accompanied by low mortality, between zero and three deaths per month, and there was no sepsis-related mortality at any point in the year. The December figures were imputed as the average of the two preceding months. Thirty-day readmission, although pre-specified as a Service-bundle component, was not operationalized during the pilot year and is therefore not reported here; this gap between definition and capture reflects the early stage of the program and is considered in the Discussion (Figure 6A-6B).

**Figure 7:**
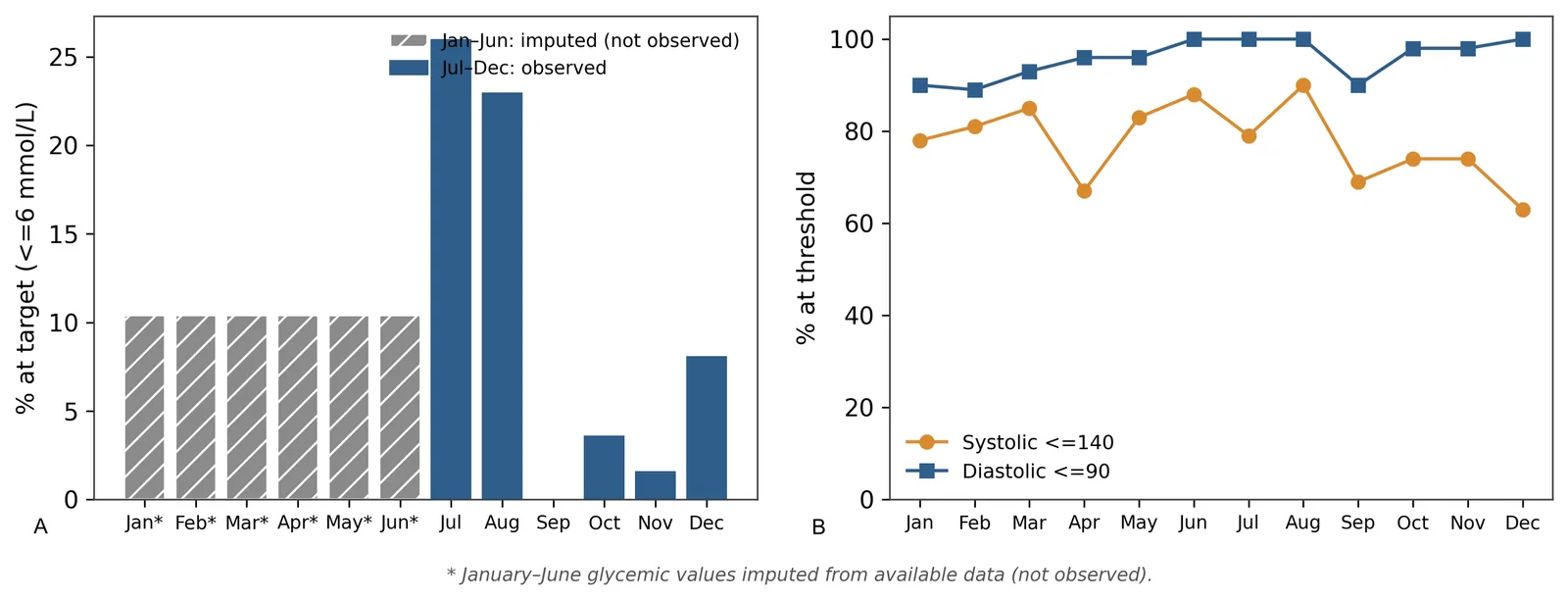
Clinical-care bundle; glycemic and blood pressure control Monthly percentage of sampled patients with diabetes at the glycemic target (≤6 mmol/L) (A), and monthly systolic (≤140 mmHg) and diastolic (≤90 mmHg) blood pressure control (B). In (A), the January-June values (hatched gray bars, months marked with an asterisk) were imputed from available data and were not observed. The July-December values (solid bars) were observed. No artificial intelligence (AI) tool was used to generate the figure.

Glycemic control, measured as the proportion of sampled patients with diabetes whose mean glucose reached the target of 6 mmol/L or below, was low, with observed second-half values ranging from 0 to 26%. Structured glycemic monitoring was operationalized only in the second half of the year, for the logistic and technical reasons noted above, so the first-half values were not prospectively collected. They are shown imputed from the available data (approximately 10%) and are marked as such in Figure 7A. The distribution of mean readings showed a substantial proportion of patients in the higher glucose ranges, prompting the involvement of the endocrine service. Blood pressure control was considerably better: systolic readings were at or below the established clinical threshold of 140 mmHg in roughly two-thirds to nine-tenths of sampled patients by month, and diastolic readings were at or below 90 mmHg in about 89 to 100% (Figure 7A-7B).

**Figure 8:**
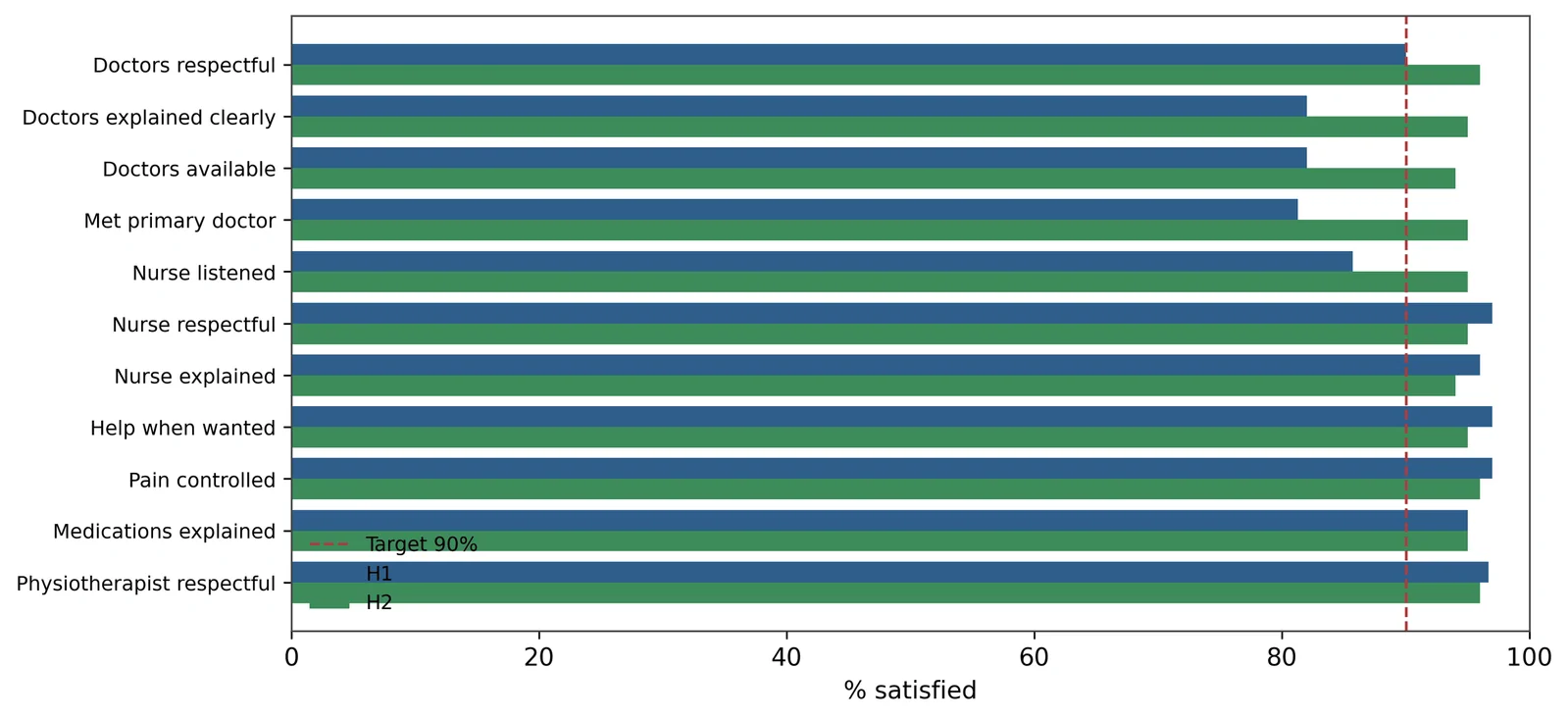
Patient-satisfaction bundle; item-level satisfaction Percentage of surveyed patients satisfied with each survey item across the physician-care, nursing-care, and service-care domains, comparing the first half (H1) and second half (H2) of the year, with a 90% reference line, applied retrospectively for orientation only. Physician-domain items showed the greatest improvement between H1 and H2. No artificial intelligence (AI) tool was used to generate the figure.

Satisfaction was high across all three domains, with item-level scores ranging from about 81% to 97%. The lowest scores in the first half of the year clustered in the physician domain, specifically the availability of doctors and the clarity of their explanations, at roughly 81 to 82%, and these rose to between 94 and 96% in the second half. The pattern illustrates how measurement at the level of individual items can localize a problem and then track its resolution (Figure 8).

**Figure 9:**
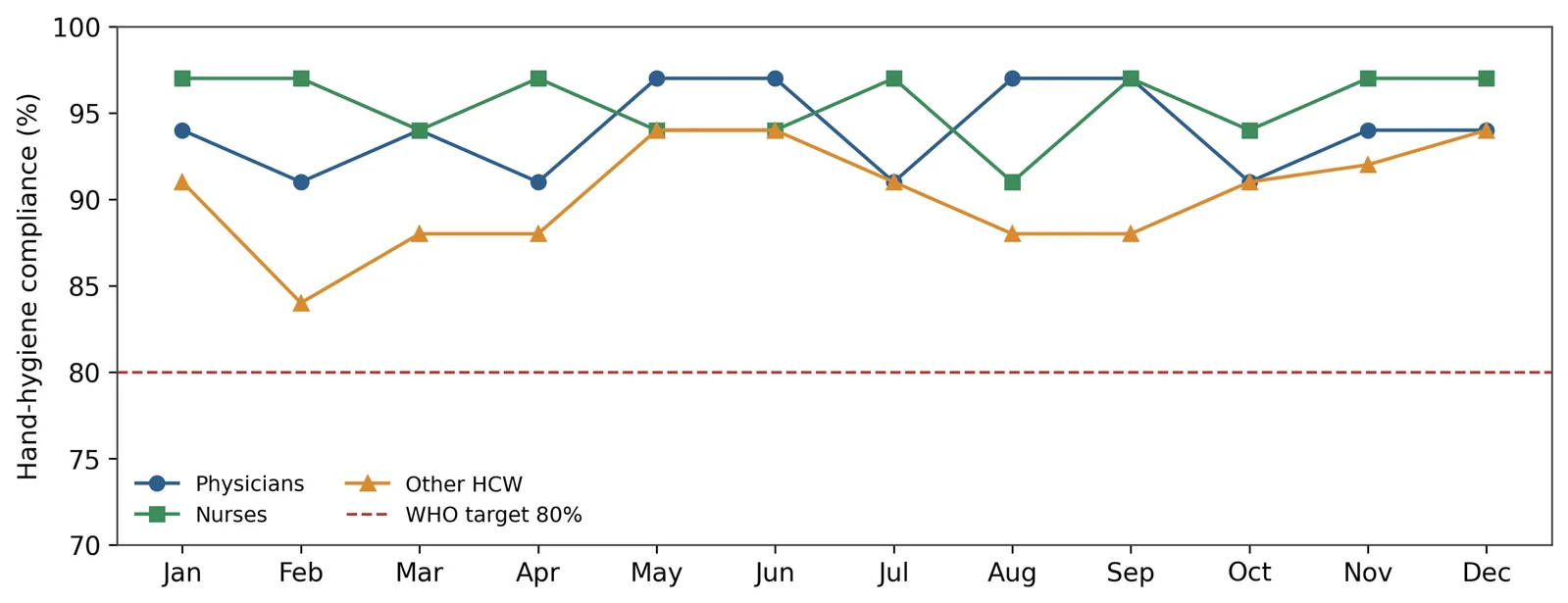
Infection-control bundle; hand-hygiene compliance Monthly hand-hygiene compliance by staff group (physicians, nurses, and other healthcare workers), audited using the WHO observation tool, with an 80% reference line applied retrospectively for orientation. There were zero MRSA, CAUTI, PICC-, and EVD-related infections during the year. MRSA: methicillin-resistant *Staphylococcus aureus;* CAUTI: catheter-associated urinary tract infection; PICC: peripherally inserted central catheter; EVD: external ventricular drain No artificial intelligence (AI) tool was used to generate the figure.

Hand hygiene compliance, audited using the World Health Organization observation methodology, was consistently high among physicians and nurses, at roughly 91-97%. At the same time, other healthcare workers formed the most variable and lowest-performing group, ranging from about 84 to 94%. No MRSA, catheter-associated urinary tract, PICC-related, or external ventricular drain-related infections occurred during the year. A transient decline in environmental measures coincided with a change in the janitorial-service provider (Figure 9).

**Figure 10:**
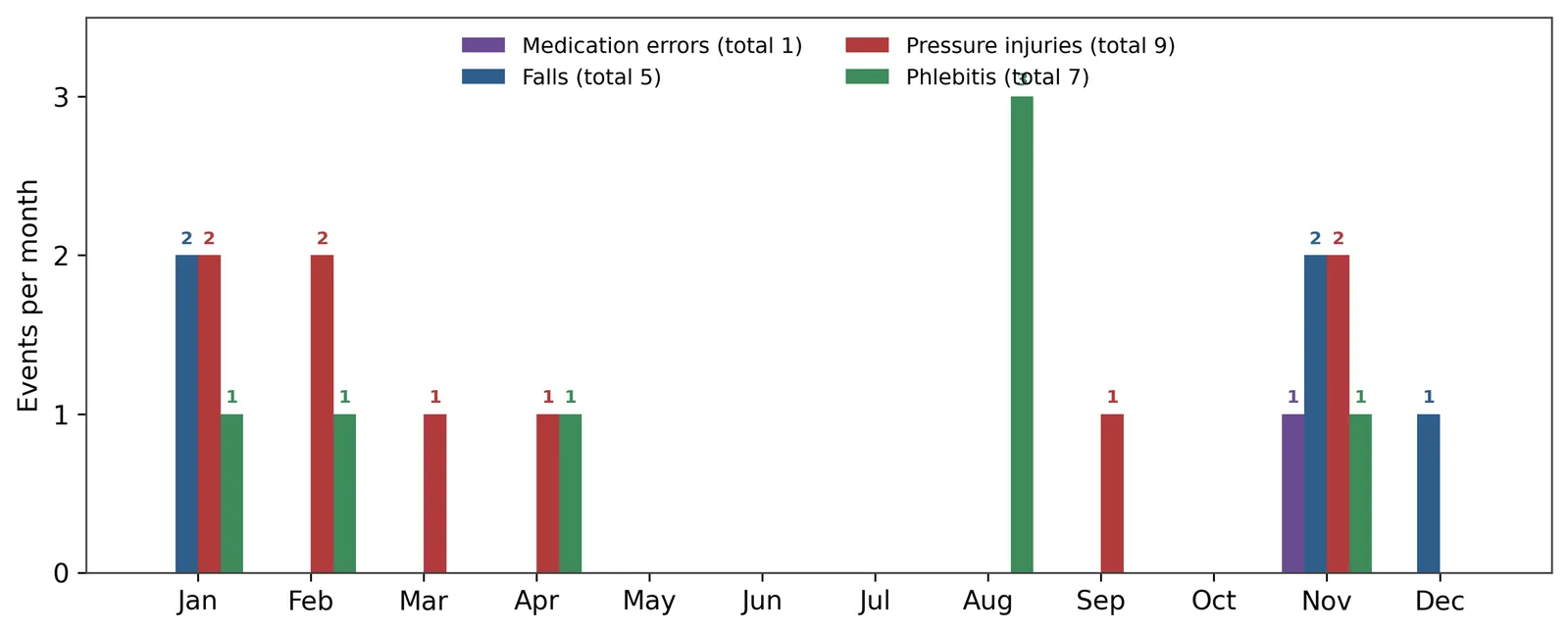
Patient-safety bundle; safety events Monthly counts of medication errors (total one), falls (total five), pressure injuries (stage 1-2; total nine), and phlebitis (total seven) during 2021. The numeral above each bar gives the count for that month, and the yearly totals appear in the legend; most months recorded no events in most categories. No artificial intelligence (AI) tool was used to generate the figure.

The absolute burden of safety events was low throughout the year. There was a single medication error, a physician prescription error recorded in November; five falls, all associated with patient confusion; nine pressure injuries, all stage 1 or 2; and seven episodes of phlebitis. Most months recorded no events in most categories (Figure 10).

## Discussion

This report shows that a neurosurgery department can build a functional quality dashboard from first principles, without electronic medical record integration or dedicated business-intelligence software, by combining clearly defined metrics, distributed but accountable data ownership, and a disciplined monthly review. The value of the approach is best seen in how readily it turned routine data into action. The timely-discharge bundle is the clearest example. Rather than a vague impression that discharges were slow, the Pareto analysis localized about three-quarters of all delays to a single addressable cause, the late writing of physician orders, and pointed directly to the remedies that followed. In the same way, item-level satisfaction data identified physician availability and communication as the weak points and then documented their improvement. In contrast, surveillance data gave the department confidence in its infection-control and safety performance and, at the same time, flagged the staff group whose hand-hygiene compliance most needed reinforcement.

Our experience both echoes and extends the existing literature. The eight-bundle structure maps closely onto the domain-based architecture of the UCLA dashboard, with its grouping of quality and safety, patient satisfaction, and efficiency and use, and onto the indicator frameworks used in national pediatric neuroscience programs, which confirms that these higher-level domains are durable organizing principles [6,9]. Where our project differs is in its starting point and its resource model. National registries and dashboards linked to the electronic record represent the mature, automated end of the spectrum [7,9]; our dashboard occupies the entry-level, manually maintained end, and it is precisely this end that is least described in the literature and most relevant to departments that lack advanced informatics. In that sense, the present report is best understood as a practical on-ramp to the kind of programs that others have already documented.

The timing of this work deserves emphasis. The dashboard was built and operated through 2021, at the peak of the COVID-19 pandemic, when the department faced acute staffing shortages, and the wider healthcare system was under crisis-level strain. The pandemic was therefore at once a confounder and a test of resilience. Its effect is evident in the data, most clearly in the contraction of elective volume and the staffing disruptions that complicated several bundles. That a department new to formal quality work could nonetheless establish a functioning dashboard, and that the dashboard continued to capture data and inform decisions throughout such a period, is itself evidence that a low-technology, role-distributed model can be created and sustained under conditions that would overwhelm a more complex system. Far from diminishing the relevance of these data, this context is part of what makes the experience worth reporting. We also acknowledge the interval between data collection in 2021 and the present report. The initiative was designed, implemented, and its data compiled during that pandemic year, but the formal write-up was deferred while the department absorbed the sustained service pressures of the pandemic and its aftermath. We have returned to the material now for two reasons: the department is drawing on this experience to reactivate and extend the program, and the contribution of the work is methodological rather than time-sensitive. The value of the report lies in a transferable, low-resource approach to building a dashboard, not in outcomes tied to a particular year. The findings are therefore presented as a methodological guide rather than as a report of current performance.

Several limitations should be acknowledged. This is a single-center descriptive report without a control group or formal statistical comparison, so the data illustrate the instrument rather than establish cause and effect. Manual data entry carries a risk of transcription error and imposes a continuing workload, and the richness of the data varies from one bundle to another. Some measures relied on sampling rather than complete capture, and rare events such as device-related infections and safety incidents occurred too infrequently to support inferential analysis. A few values were incomplete: the December service figures were imputed from adjacent months, and the first-half glycemic results were imputed from the available data. Thirty-day readmission and first-half glycemic monitoring are the clearest examples: the former was defined but not captured, and the latter began only in the second half of the year. More broadly, not every pre-specified indicator was operationalized in the first year. Fixing definitions in advance ensured that the meaning of each measure was stable and reproducible, but capturing every defined component is a function of program maturity that we had not yet fully achieved; 30-day readmission is the clearest example. We wish to be candid that two of these limitations are more than generic features of an early program. First, a pre-specified core indicator, 30-day readmission, was not collected at any point during the study year, so the Service bundle was only partially realized rather than merely imperfectly measured. Second, the benchmarks used to characterize performance throughout the Results were chosen after the data had been seen, which means those characterizations are orientational rather than evaluative and are vulnerable to post-hoc rationalization. We therefore caution against reading the Results as an assessment of whether the department met defined standards; they are better understood as a demonstration of what the dashboard can surface. The remaining constraints, namely the imputation of some values, reliance on sampling, low frequency of certain events, and absence of statistical comparison, are characteristic of an early, manually maintained program and define a clear path toward maturity through progressive automation, integration with hospital information systems, the setting of explicit prospective targets, and formal, adequately powered evaluation of specific interventions.

These constraints are characteristic of an early, manually maintained quality program, and they define a clear path toward maturity through progressive automation, integration with hospital information systems, setting of explicit prospective targets, and the formal, adequately powered evaluation of specific interventions. A further limitation concerns our use of benchmarks. The operational thresholds used for orientation (for example, 80% hand-hygiene compliance, approximately 85% bed occupancy, and 90% item-level satisfaction) were selected retrospectively rather than set in advance, and they therefore function as illustrative reference points rather than pre-specified targets. Judging a full year of results against thresholds chosen after the data were seen invites post-hoc rationalization, because a given result can be framed against whichever reference it happens to clear. For this reason, we have reported performance descriptively throughout and have avoided characterizing any bundle as having met or missed a target. The established clinical thresholds used in the Clinical Care bundle (systolic blood pressure ≤140 mmHg, diastolic ≤90 mmHg, and pre-meal glucose ≤6 mmol/L) are externally defined and are not subject to this concern. Setting prospective, pre-specified targets for the operational bundles is a priority as the program matures.

The principal contribution of this report is its generalizability. The eight-bundle template, the metric definitions in Table 1, and the front-liner and back-up model of data ownership are offered so that another neurosurgery department, particularly one in a resource-limited setting or at the beginning of its quality journey, can adopt or adapt them directly.

## Conclusions

A neurosurgery quality dashboard can be built from first principles, maintained by hand, and run with modest resources when its metrics are clearly defined, its data ownership is assigned, and its review is disciplined. In our single-center experience, even for a team new to quality work and through the strain of the COVID-19 pandemic, an eight-bundle dashboard reliably surfaced and prioritized actionable problems and prompted targeted interventions. We do not, however, demonstrate that those interventions improved outcomes, as the report includes no post-intervention follow-up or statistical comparison, and the program was disciplined in its definitions but still developing in its data capture. The dashboard's value lies in making departmental performance visible and directing attention to where improvement is needed, with measurable outcome improvement reserved for the prospective evaluation we plan. We offer this eight-bundle structure as a replicable, patient-centered model for departments beginning to measure and improve their care.

## References

[1] Dagi TF, Dagi LR (2019). Commentary: Ernest Codman and the impact of quality improvement in neurosurgery: a century since the idea of the "end result". Neurosurgery.

[2] Kohn LT, Corrigan JM, Donaldson MS (2000). To Err Is Human: Building a Safer Health System. https://www.simlaweb.it/wp-content/uploads/2019/07/to_err_is_human.pdf.

[3] McLaughlin N, Afsar-Manesh N, Ragland V, Buxey F, Martin NA (2014). Tracking and sustaining improvement initiatives: leveraging quality dashboards to lead change in a neurosurgical department. Neurosurgery.

[4] Weprin BE (2018). On developing the tools and metrics for a neurosurgical quality program. Quality and Safety in Neurosurgery.

[5] Richards S (2026). Neurosurgical outcomes and quality metrics. What healthcare leaders need to know. https://www.europeanhhm.com/surgical-speciality/neurosurgical-outcomes-and-quality-metrics.

[6] (2026). UCLA department of neurosurgery: value-based neurosurgery report. https://www.uclahealth.org/sites/default/files/documents/2015-Value-Based-Neurosurgery.pdf.

[7] Parker SL, McGirt MJ, Bekelis K (2015). The National Neurosurgery Quality and Outcomes Database Qualified Clinical Data Registry: 2015 measure specifications and rationale. Neurosurg Focus.

[8] Azadmanjir Z, Sadeghi-Naini M, Dashtkoohi M, Moradi-Lakeh M, Arabkheradmand J, Harrop JS, Rahimi-Movaghar V (2024). The design of a quality improvement dashboard for monitoring spinal cord and column injuries. Inform Med Unlocked.

[9] (2026). NHS England: pediatric neurosciences (neurosurgery) quality dashboard 2020/21. https://www.england.nhs.uk/wp-content/uploads/2021/05/Paediatric-Neurosciences-Neurosurgery-Quality-Dashboard-2021-22.pdf.

[10] Khan A, Naushad Chaudhry M, Khalid S, Nandi D (2014). Improvement of patient satisfaction with the neurosurgery service at a large tertiary care, London-based hospital. BMJ Qual Improv Rep.

[11] Feller CN, Bodenbach EM, Kolinski JM, Sinson GP (2024). Improving quality metrics in neurosurgery: a spinal surgery 3-year case review. Neurosurg Pract.

[12] McLaughlin N, Jin P, Martin NA (2015). Assessing early unplanned reoperations in neurosurgery: opportunities for quality improvement. J Neurosurg.

[13] Rolston JD, Han SJ, Parsa AT (2015). Quality improvement in neurosurgery. Neurosurg Clin N Am.

[14] Steiger HJ, Uhl E (2001). Risk Control and Quality Management in Neurosurgery. Acta Neurochir Suppl, Vol 78.

[15] Berger MS, Wachter RM, Greysen SR, Lau CY (2013). Changing our culture to advance patient safety: the 2013 AANS presidential address. J Neurosurg.

[16] (2026). World Health Organization: WHO guidelines on hand hygiene in health care. https://www.who.int/publications/i/item/9789241597906.

[17] Langley GL, Moen R, Nolan KM, Nolan TW, Norman CL, Provost LP (2009). The Improvement Guide: A Practical Approach to Enhancing Organizational Performance. https://healthleadsusa.org/wp-content/uploads/2019/11/Health-Leads-PDSA-Worksheet.pdf.

